# Metabolic bariatric surgery pays off: a longitudinal analysis of weight loss and HbA1c changes in real-world patients data in the West of Scotland

**DOI:** 10.1038/s41366-025-01956-6

**Published:** 2025-11-19

**Authors:** Beatrice Leyaro, Lyz Howie, Abdulmajid Ali, Raymond Carragher

**Affiliations:** 1 School of Computing, Engineering and Physical Sciences, University of the West of Scotland, Paisley, United Kingdom; 2 School of Public Health: Epidemiology and Biostatistics Department, KCMC University, Moshi, Tanzania; 3 School of Health and Life Sciences, University of the West of Scotland, Lanarkshire, United Kingdom; 4 Department of General & Upper GI Surgery, University Hospital Ayr, Ayr, United Kingdom

**Keywords:** Epidemiology, Weight management

## Abstract

**Rationale:**

The long-term trajectories of weight loss and glycemic outcomes for patients who undergo bariatric surgery remain underexplored, particularly when comparing individuals who undergo surgery to those who are eligible but do not proceed after referral.

**Methods:**

This retrospective cohort study examined 411 patients with type 2 diabetes and obesity who were referred for consideration of metabolic bariatric surgery (MBS) at University Hospital Ayr, Ayrshire & Arran, between January 2009 and December 2020. The primary outcomes were change in percentage total weight loss (%TWL) and Glycosylated Hemoglobin (HbA1c) from baseline to five years.

**Results:**

Of the 411 patients included, 225 (54.7%) did not undergo bariatric surgery. There were no significant differences between the surgery and non-surgery groups with respect to age, BMI or sex. 260 (63%) of the patients were female, the mean age of patients was 48.3 years (SD = 8.5), and the mean BMI was 47.4 kg/m² (SD = 7.9). At five years, patients who underwent surgery maintained a 22.0% TWL reduction compared to 8.6% in the non-surgery group (*p* < 0.001). HbA1c decreased by 1.0% (95% CI: −1.31, −0.70) in the surgery group but increased by 0.4% (95% CI: 0.09, 0.71) in the non-surgery group. Adjusted mixed-effects modelling showed the annual HbA1c level decreased by 0.13% (95% CI: −0.18, −0.07; *p* < 0.001) in the surgery group, compared to an increase of 0.11% (95% CI: 0.05, 0.17; *p* < 0.001) in the non-surgery group. %TWL decreased by 1.31% per year (95% CI: −1.73, −0.88; *p* < 0.001) in the surgery group, while the non-surgery group experienced an increase of 1.11% (95% CI: 0.66, 1.55; *p* < 0.001).

**Conclusion:**

Patients eligible for MBS who proceed with surgery achieve superior weight loss and glycaemic control compared to those who decide not to proceed with surgery. Opting out of surgery may have significant health implications, highlighting the need for alternative interventions such as intensive lifestyle modification, pharmacotherapy, and diabetes education programs for those unable or unwilling to undergo MBS.

## Introduction

The global burden of obesity and type 2 diabetes mellitus (T2DM) continues to rise, posing substantial challenges to healthcare systems worldwide [1, 2]. The global prevalence of obesity nearly doubled between 1990 and 2022, with approximately 2.5 billion adults classified as overweight, and over 890 million adults classified as living with obesity in 2022 [3]. In 2024, according to the Diabetes Atlas [4] which synthesised data from 193 sources across 109 countries, an estimated 589 million adults aged 20–79 years were living with diabetes globally, with about 43% undiagnosed.

In the United Kingdom (UK), adult obesity has increased significantly, rising from 20.1% in 2014 to 29% in 2019, and a recent predictive modelling study projects that this figure will reach 36% by 2040 [5]. Obesity is a well-established risk factor for the development and progression of T2DM, and individuals with both conditions are at increased risk of cardiovascular disease, cancer, renal dysfunction, and premature mortality [4, 6]. The co-existence of obesity and diabetes poses a significant health challenge and complicates their management. Traditional approaches to managing obesity and T2DM, including lifestyle modification and pharmacotherapy, are not sufficient to achieve and maintain weight loss and glucose control [7–9].

Metabolic and bariatric surgery has emerged as a highly effective treatment modality for achieving substantial and sustained weight loss, along with significant improvement in glycemic control, among individuals with obesity and T2DM [10–14], with several randomized controlled trials and observational studies demonstrating superior outcomes in weight reduction and glycemic control among patients undergoing metabolic bariatric surgery (MBS) compared to those receiving standard medical therapy [15–17]. A large UK-based cohort study also demonstrated the real-world effectiveness of bariatric surgery in achieving significant long-term weight loss and reduction in obesity-related comorbidities such as type 2 diabetes, hypertension, and cardiovascular disease [18]. Despite such evidence, significant variation in the application of, and an unmet need for, metabolic surgery persists. In comparison to other countries, the UK has been slower in uptake of metabolic bariatric surgery, with 0.20% of the eligible population, around 3.21 million people, undergoing obesity surgery, with regional variation in rates ranging from 0.08% to 0.41% [19, 20]. Several studies have identified barriers contributing to patient drop-out before surgery, and there is a need to understand outcomes in those who do not receive surgical intervention despite referral [19, 21, 22].

While studies have reported improved outcomes following bariatric surgery in real-world clinical settings, the evidence regarding long-term weight and metabolic trajectories such as HbA1c changes in patients referred to bariatric surgery, but who do not proceed to surgery, remains limited, particularly in diverse unselected populations [18, 23, 24]. In addition, existing clinical guidelines and studies continue to emphasize the importance of perioperative support, yet little is known about the long-term outcomes in routine practice, particularly for those who opt for non-surgery after referral, given that individuals with severe obesity who do not undergo bariatric surgery are characterized by a high burden of disease and impaired health status [25, 26].

This study aims to address a knowledge gap in the literature by using longitudinal real-world patient data to investigate long-term weight and HbA1c changes among patients with obesity and T2DM who were referred for bariatric surgery. Specifically, the study compares outcomes between individuals who underwent metabolic bariatric surgery and those who were referred, but did not proceed with surgery, to evaluate the benefit of metabolic bariatric surgery over a five-year period. By examining both groups over time, the study gives insight into the beneficial effects of metabolic bariatric surgery, while also informing care options available for those who opt not to undergo surgery. Such evidence is essential for understanding the effectiveness and durability of metabolic bariatric surgery in routine clinical practice. This study uses routinely collected clinical data from the University Hospital Ayr in the West of Scotland to assess these long-term outcomes in both surgical and non-surgical patients eligible for bariatric intervention.

## Methods

### Study design

This was a retrospective cohort study of prospectively collected data of patients with obesity (BMI ≥35 kg/m²) and T2DM who were referred to the Bariatric and Metabolic Department, University Hospital Ayr, between January 2009 and December 2020, for metabolic bariatric surgery consideration.

### Data sources and data collection

The study utilized routinely collected clinical data extracted from multiple sources including an electronic bariatric database in the Bariatric and Metabolic Department of the University Hospital Ayr, the Scottish Care Information (SCI) Diabetes platform (SCI-DC, Scotland) [27], Clinical Portal [28], and bariatric patients’ assessment forms (a non-electronic database), previously described by Leyaro et al. [14]. Briefly, extracted baseline data included sociodemographic variables such as age, sex of the patients, whether patients received surgery or not, comorbidities such as hypertension, deprivation quintile from the Scottish Index of Multiple Deprivation (SIMD) [29], sleep apnea and other clinical parameters, anthropometric measurements such as weight and height, diabetes diagnosis date, and laboratory data related to glycemic control (Glycosylated Hemoglobin (HbA1c)). Follow-up data on weight and HbA1c were also obtained from these databases. The routine follow-up appointments for patients who have received a bariatric procedure is usually scheduled at 6 months, and 12 months postoperatively, and then every 12 months thereafter for at least two years [30]. The follow up data for the subsequent years for both groups were extracted from the SCI Diabetes platform. In case of missing data on weight or HbA1c in the bariatric database, this information was obtained from the Clinical Portal or SCI Diabetes, where present.

### Inclusion criteria

For the current analysis, all adults aged 18 years and above who were referred to the Department of Bariatric Metabolic surgery at University Hospital Ayr and either received a bariatric procedure, or did not receive surgery, were included. In this study individuals who did not receive metabolic bariatric surgery (non-surgery group) are those patients who began the bariatric surgery process by attending an initial evaluation visit in the clinic but did not proceed to surgery. These patients may have disengaged at any point after the initial visit, including after months of participation up to or even after scheduling a surgery date.

### Exclusion criteria

Metabolic surgery patients with less than 1 year of postoperative follow-up were excluded. Patients who did not receive metabolic surgery were excluded if they had less than 1 year follow-up. Patients with missing information on baseline weight and HbA1c were excluded, as were patients who received bariatric procedures outside University Hospital Ayr, and patients who underwent revision or modification procedures.

### Outcome measures

Weight change was assessed using two established measures from the literature [31–33]. First percentage total weight loss (%TWL) calculated as (Initial Weight − Follow-up Weight)/Initial Weight * 100 and second, the probability of achieving at least 20% total weight loss, a threshold which was used to define an optimal clinical response weight loss based on previous studies [32]. The initial weight in this context refers to the referral weight, the weight recorded at the time of the patient’s referral.

Changes in glycemic control were assessed using HbA1c levels. Specifically, the change in HbA1c over time was determined by calculating the difference between the referral HbA1c measurement and HbA1c values recorded at selected time points during the five-year follow-up period (at 1, 2, 3, 4 and 5 years). Additionally, a binary HbA1c variable was created using two sets of clinical cutoffs based on criteria defined by the American Diabetes Association (ADA): (i) HbA1c < 6.0% vs. ≥6.0% and (ii) HbA1c < 6.5% vs. ≥6.5% [34]. These thresholds align with the diabetes remission criteria reported by Brethauer et al. [31].

### Statistical data analysis

All statistical analyses were performed using Stata, version 18.0; Stata Corp. Descriptive statistics were calculated for baseline characteristics. Frequency and percentage (%) were reported for qualitative data, and the mean, median and measures of dispersion (standard deviation, confidence intervals, and interquartile range) for quantitative data. All study variables were comparatively analysed between patients who received metabolic bariatric surgery and those who did not. *t*-tests were used to compare normally distributed continuous variables (such as age), while Kruskal–Wallis tests were applied for non-normally distributed variables (such as duration of diabetes). Chi-square tests were used to examine group differences in categorical variables.

To assess the probability of achieving the optimal clinical response weight loss outcomes i.e. achieving ≥20% TWL at five years, modified Poisson regression modelling was applied. Prevalence ratios and their corresponding 95% confidence intervals were reported.

A repeated measures linear mixed model was fitted to examine %TWL and HbA1c change over time. The model included fixed effects terms for time, group (surgery vs. non-surgery), and their interaction (time × group). Covariates included age, sex, deprivation quintiles, baseline BMI, and duration of diabetes. A random intercept and random slope for time at the participant level (participant id) was included to account for multiple observations over time. The estimated mean %TWL and HbA1c %, coefficients, standard error, and 95% confidence intervals (CIs) were reported. A significance level of *p* < 0.05 was used throughout. Linearity and homoscedasticity assumptions of the linear mixed model were evaluated by inspecting the residuals versus fitted values plot. Visual assessment did not reveal substantial deviations from linearity or evidence of heteroscedasticity.

### Ethical approval

Ethical approval was granted by South Central-Hampshire A Research Ethics Committee, REC reference: 22/SC/0228. The National Health Service (NHS) Ayrshire and Arran research and development management unit provided permission to access and use the data. Informed consent was not applicable for this study since it was a retrospective cohort study of routinely collected data and no direct contact with the patients was attempted. The study adhered to ethical guidelines to maintain patient confidentiality and data integrity.

## Results

### Participant characteristics

A total of 555 patients with obesity and diabetes who were referred for consideration of metabolic bariatric surgery were assessed for eligibility for inclusion in the analysis, with 411 meeting the inclusion criteria. Of these, 186 (45.3%) underwent bariatric surgery. The median time to surgery from referral date was 9.9 months (IQR: 7.2, 15.2). At baseline the patients had a mean age of 48.3 years (SD = 8.5), a mean BMI of 47.4 kg/m² (SD = 7.9), and 260 (63.3%) were female. Compared to patients who receive surgery, the median duration of diabetes was significantly shorter for those who did not receive surgery: 3.0 years (IQR: 1.1, 7.7) versus 5.0 years (IQR: 2.0, 9.0) (*p* < 0.001). The prevalence of depression was 48.4% among patients who received bariatric surgery compared to 62.1% among those who did not. This difference was statistically significant (*p* = 0.024). All other characteristics such as age, initial BMI, and HbA1c % were similar between the groups (Table 1).

**Table 1:** Baseline characteristics of the patients who had bariatric surgery compared to those who did not (*n* = 411).

Variable	All (*n* = 411)	Surgery group (186)	Non-surgery group (225)	*P*-value
Age (years), mean (SD)	48.3 (8.5)	49.0 (8.1)	47 (8.7)	0.095
Duration of diabetes (years) median (IQR)	4 (1.7–8.4)	5.0 (2.0–9.0)	3.0 (1.1–7.7)	<0.001^a^
Sex				
Male (%)	151 (36.7)	64 (34.4)	87 (38.7)	0.373
Female (%)	260 (63.3)	122 (65.6)	138 (61.3)	
Referral BMI, mean (SD)	47.4 (7.9)	47.1 (8.1)	47.6 (7.7)	0.473
Referral HbA1c, mean (SD)	7.8 (1.5)	7.7 (1.6)	7.8 (1.5)	0.316
BMI Obesity Classes				0.811
Class 1 (30 to < 35) (%)	9 (2.2)	5 (2.7)	4 (1.8)	
Class 2 (35 to < 40) (%)	59 (14.4)	26 (14.0)	33 (14.7)	
Class 3 (≥40) (%)	343 (83.4)	155 (83.4)	188 (83.5)	
Insulin use				
No (%)	336 (81.8)	155 (83.3)	181 (80.4)	0.450
Yes (%)	75 (18.2)	31 (16.7)	44 (19.6)	
Other comorbidities				
Hypertension				0.332
No (%)	157 (53.2)	103 (55.4)	54 (49.5)	
Yes (%)	138 (46.8)	83 (44.6)	55 (50.5)	
Missing = 116				
Depression				
No (%)	137 (46.6)	96 (51.6)	41 (37.9)	0.024^a^
Yes (%)	157 (53.4)	90 (48.4)	67 (62.1)	
Missing = 117				
Sleep apnoea				0.712
No (%)	339 (82.5)	152 (81.7)	187 (83.1)	
Yes (%)	72 (17.5)	34 (18.3)	38 (16.9)	
Deprivation quintile				0.313
SIMD 1 (most deprived) (%)	177 (43.1)	74 (39.8)	103 (45.9)	
SIMD 2 (%)	91 (22.2)	41 (22 1)	50 (22.3)	
SIMD 3 (%)	75 (18.3)	33 (17.7)	42 (18.8)	
SIMD 4 (%)	39 (9.5)	21 (11.3)	18 (8.0)	
SIMD 5 (least deprived) (%)	28 (6.8)	17 (9.1)	11 (4.9)	
Missing = 1				

*SIMD* Scottish Index of Multiple Deprivation, *SD* Standard deviation, *BMI* Body Mass Index, *IQR* Interquartile Range, *HbA1c* Glycated hemoglobin.

^a^Significant *p*-value < 0.05.

### Proportion of patients who achieved optimal clinical response weight loss (%TWL) between surgery and non-surgery group

At five years, %TWL data were available for 229 patients, including 129 patients in the surgery group and 100 patients in the non-surgery group. A total of 86 (37.6%) achieved at least ≥20% of total weight loss at five years of follow up. Of those who achieved at least ≥20% of TWL at five years, 75 (87.2%) were among patients who underwent bariatric surgery in comparison to 11 (12.8%) for the non-surgery group, and the difference was statistically significant (*p* < 0.05) (Supplementary Fig. S1).

### Prevalence ratio for achieving optimal clinical response weight loss at five years

A multivariable modified Poisson regression model was fitted to estimate the prevalence ratio of achieving optimal clinical response weight loss at five years. Adjusted for age, sex, deprivation status, pre-surgery BMI, and duration of diabetes, the estimated proportion achieving ≥20% TWL is 59.4% (95% CI: 50.97, 67.84) for patients who received bariatric surgery, in comparison to 10.8% (95% CI 4.78, 16.83) for the non-surgery group. Patients who received bariatric surgery were 5.49 times more likely to achieve ≥20% TWL compared to the non-surgery group, and the difference was statistically significant (PR = 5.49, 95% CI: 3.08, 9.89; *p* < 0.001) (Supplementary Table S1).

### Linear mixed model for percentage total weight loss estimates

**Fig. 1:**
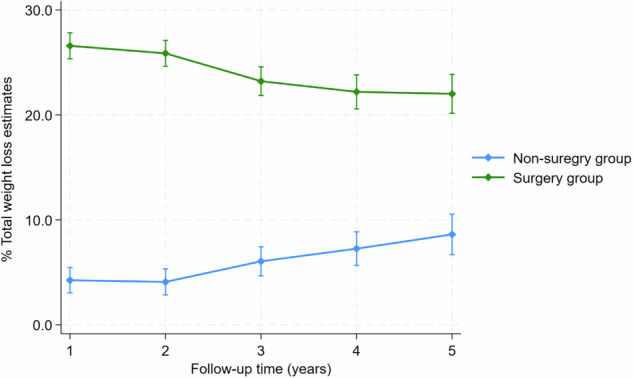
Differences in estimated weight changes between surgery and non-surgery group across different time points. Line plot showing mean estimated %TWL with 95% confidence intervals, from year 1 to year 5 in the surgery group (green line) and non-surgery group (blue line). Mean %TWL estimates from year 1 to 5 show a decreasing trend in weight loss after an initial peak in the surgery group while the non-surgery group shows an increase in weight loss over time.

A linear mixed model was applied to model percentage total weight loss over five years (Table 2). The model was adjusted for time, surgery group, sex, duration of diabetes, age prior to surgery, and deprivation status. The variability between participants accounted for 76.1% of the total variance in the model’s random effects. The interaction between time and treatment groups (surgery versus non-surgery) had a significant negative coefficient (−2.43; 95% CI: −3.04, −1.81; *p* < 0.001) indicating that the rate of weight loss over time was significantly different between patients who received surgery and those who did not. Patients in the surgery group had an average annual decrease in weight loss of −1.31% (95% CI: −1.73, −0.88), whereas those in the non-surgery group had an annual increase in weight loss of 1.11% (95% CI: 0.66, 1.55) (Fig. 1).

**Table 2:** Multivariable mixed model for weight loss.

Characteristics	Coefficient (β)	SE	95% CI	*P*-value
Fixed effects				
Intercept	0.18	3.57	(−6.83, 7.20)	0.959^a^
Treatment group				
Non-surgery group	Ref			
Surgery group	25.28	0.98	(23.35, 27.21)	<0.001^a^
Time (years)	1.11	0.23	(0.66, 1.55)	<0.001^a^
Treatment typeΧtime	−2.43	0.31	(−3.04, −1.81)	<0.001^a^
Sex				
Female	Ref			
Male	0.56	0.84	(−1.08, 2.21)	0.503
Age pre-surgery (years)	−0.14	0.05	(−0.24, −0.04)	0.005^a^
Pre-surgery BMI	0.17	0.05	(0.69, 0.27)	0.001^a^
Duration of diabetes (years)	0.11	0.08	(−0.05, 0.27)	0.182
SIMD quintile				
SIMD 1 = Most deprived	Ref			
SIMD 2	−0.11	1.05	(−2.17, 1.96)	0.919
SIMD 3	1.76	1.12	(−0.43, 3.96)	0.115
SIMD 4	0.48	1.42	(−2.31, 3.28)	0.733
SIMD 5 = Least deprived	1.11	1.65	(−2.13, 4.35)	0.501
Random effects	Variance			
Participant	67.26			
Time(years)	5.73			
Residual	15.42			

Adjusted for Time, age, sex, SIMD quintiles, pre-surgery BMI and duration of diabetes.

*SIMD* Scottish Index of Multiple Deprivation, *SE* Standard error, *BMI* Body Mass Index.

^a^Significant *p*-value < 0.05.

There was a significant positive association between pre-surgery BMI and %TWL. Adjusted for time, surgery group, sex, duration of diabetes, age prior to surgery, and deprivation status, the estimated effect for pre-surgery BMI was 0.17 (95% CI: 0.69, 0.27; *p* < 0.001). A unit increase in pre-surgery BMI corresponds to 0.17 increase in %TWL on average. There was a significant negative association between age and %TWL, with a year increase in age pre-surgery decreasing %TWL by 0.14 (95% CI: −0.24, −0.04; *p* = 0.005). Duration of diabetes, deprivation quintiles, and sex were not significantly associated with %TWL.

### Estimated adjusted mean %TWL, comparison between surgery and non-surgery group over time

The adjusted estimated change in %TWL across different times point is presented in Supplementary Table S2. Overall, the surgery group had a significantly higher %TWL compared to the non-surgery group at all time points. The mean %TWL was higher for the surgery group at 1 year and 2 years, 26.5% (95% CI: 25.3, 27.8) and 25.8% (95% CI: 24.6, 27.0) respectively, afterward there was a decrease from years 3 to five. For the non-surgery group, the mean %TWL was lower at 1 year and 2 years, followed by a steady increase from year 3 to year 5 (Fig. 1).

### Proportion of patients achieving threshold of HbA1c of <6.0% or <6.5 at five years

**Fig. 2:**
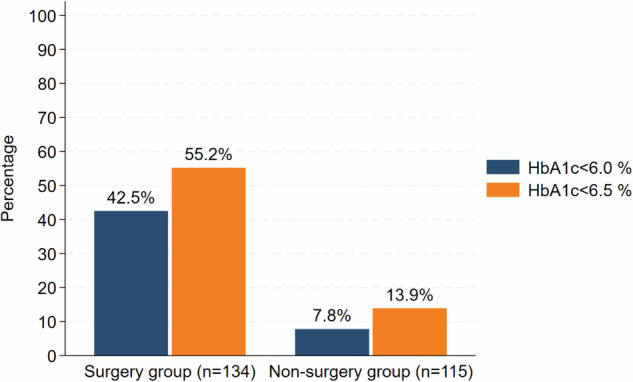
Proportion of patients who achieved HbA1c of <6.0% or <6.5 at five years. Bar chart showing the percentage of patients who achieved HbA1c of <6.0% (navy bars) and <6.5% (orange bars) at five years in surgery group (n = 134) and non-surgery group (n = 115). The surgery group shows substantially higher proportions of patients reaching both glycaemic thresholds compared to the non-surgery group.

At five years, HbA1c data were available for 249 patients, including 115 patients in the non-surgery group and 134 in the surgery group. Among patients with available HbA1c data at five years 57 (42.5%) of the patients in the surgery group had an HbA1c < 6.0% compared to 9 (7.8%) in the non-surgery group. When the threshold of HbA1c was set at <6.5%, at five years 74 (55.2%) of the patients in the surgery group achieved this threshold compared to 16 (13.9%) in the non-surgery group. The difference was statistically significant (*p* < 0.001) (Fig. 2).

### Linear mixed model for HbA1c change estimates

A linear mixed model was fitted to examine HbA1c change over a five-year period (Table 3). The model was adjusted for time, treatment group, sex, age prior to surgery, deprivation status, and duration of diabetes. The variability between participants accounted for 53.8% of the total variance in the model’s random effects.

**Table 3:** Multivariable mixed model for HbA1c change.

Characteristics	Coefficient (β)	SE	95% CI	*P*-value
Fixed effects				
Intercept	9.55	0.53	(8.51, 10.59)	<0.001^a^
Treatment group				
Non-surgery group	Ref			
Surgery group	−0.74	0.15	(−1.03, −0.44)	<0.001^a^
Time (years)	0.11	0.03	(0.06, 0.17)	<0.001^a^
Treatment typeΧtime	−0.24	0.041	(−0.32, −0.16)	<0.001^a^
Sex				
Female	Ref			
Male	0.17	0.125	−(0.07, 0.42)	0.161
Age pre-surgery (years)	−0.02	0.007	(−0.03, −0.01)	0.024^a^
Pre-surgery BMI	−0.03	0.007	(−0.05, −0.01)	<0.001^a^
Duration of diabetes (years)	0.07	0.01	(0.05, 0.09)	<0.001^a^
SIMD quintile				
SIMD 1 = Most deprived	Ref			
SIMD 2	−0.13	0.15	(−0.44, 0.17)	0.396
SIMD 3	−0.10	0.16	(−0.43, 0.22)	0.533
SIMD 4	0.106	0.21	(−0.32, 0.53)	0.623
SIMD 5 = Least deprived	−0.34	0.24	(−0.083, 0.15)	0.170
Random effects	Variance			
Participant	1.26			
Time(years)	0.075			
Residual	1.013			

Adjusted for Time, age, sex, SIMD quintiles, pre-surgery BMI and duration of diabetes.

*SIMD* Scottish Index of Multiple Deprivation, *SE* Standard error, *BMI* Body Mass Index.

^a^Significant *p*-value < 0.05.

The model demonstrated a significant negative interaction between time and treatment (surgery versus non-surgery group) (−0.24; 95% CI: −0.32, −0.16; *p* < 0.001). The coefficient of the interaction terms indicates that the surgery group experienced a significantly decreasing rate of HbA1c over time compared to the non-surgery group.

There was a significant negative association between pre-surgery BMI and HbA1c. Adjusted for time, treatment group, sex, age prior to surgery, deprivation quintile, and duration of diabetes, the estimated coefficient for pre-surgery BMI was −0.03 (95% CI: −0.05, −0.01; *p* < 0.001), indicating that for each unit increase in pre-surgery BMI there is a decrease in HbA1c of 0.03 units, on average. There was a significant positive association between duration of diabetes and HbA1c, with each one-year increase in diabetes duration associated with a 0.07% increase in HbA1c (95% CI: 0.05, 0.09; *p* < 0.001), after adjusting for other variables in the model. Age showed a significant negative association with HbA1c, with each additional year of age prior to surgery associated with a 0.02% decrease in HbA1c (95% CI: −0.03, −0.01; *p* = 0.024).

### Estimated HbA1c change and mean difference between surgery and non-surgery over time

The change in HbA1c over time was significantly different between the surgery group and non-surgery group across the follow-up times. At 1 year the surgery group had a mean HbA1c of 6.02% (95% CI: 5.82, 6.23) compared to 7.84% (95% CI: 7.65, 8.04) in the non-surgery group, with a mean difference of −1.82 (95% CI: −2.10, −1.53; *p* < 0.001). This significant difference persisted throughout the follow-up period. By Year 3 the surgery group maintained a low HbA1c of 6.43% (95% CI: 6.19, 6.67), whereas the non-surgery group had increased to 8.37% (95% CI: 8.14, 8.60), with a mean difference of −1.93 (95% CI: −2.26, −1.61; *p* < 0.001). At Year 5 the surgery group showed sustained HbA1c reduction of 6.69% (95% CI: 6.39, 6.98), while the non-surgery group reached 8.27% (95% CI: 7.97, 8.57), with a mean difference of 1.58 (95% CI: −2.01, −1.16; *p* < 0.001) (Table 4).

**Table 4:** Estimated mean change and mean difference in HbA1c between surgery and non-surgery.

Time	Surgery group	Non-surgery group	Mean difference	
	Estimate (95% CI)	Estimate (95% CI)	Estimate (95% CI)	*P*-value
Baseline	7.70 (7.49, 7.91)	7.89 (7.70, 8.08)	−0.19 (−0.47, 0.08)	0.180
Year 1	6.02 (5.82, 6.23)	7.84 (7.65, 8.04)	−1.82 (−2.10, −1.53)	<0.001^a^
Year 2	6.19 (5.97, 6.40)	8.23 (8.02, 8.44)	−2.04 (−2.34, −1.74)	<0.001^a^
Year 3	6.43 (6.19, 6.67)	8.37 (8.14, 8.60)	−1.93 (−2.26, −1.61)	<0.001^a^
Year 4	6.56 (6.30, 6.82)	8.35 (8.10, 8.62)	−1.79 (−2.15, −1.42)	<0.001^a^
Year 5	6.69 (6.39, 6.98)	8.27 (7.97, 8.57)	−1.58 (−2.01, −1.16)	<0.001^a^

^a^Significant *p*-value < 0.05.

**Fig. 3:**
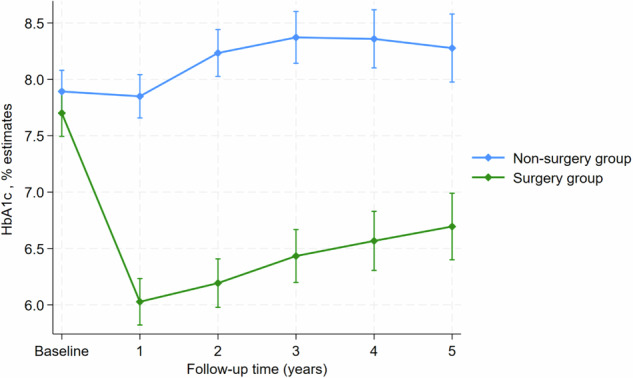
Differences in estimated HbA1c between surgery and non-surgery group across different time points. Line plot showing mean HbA1c estimates with 95% confidence intervals from baseline to year 5 in the surgery group (green line) and non-surgery group (blue line). Lower HbA1c levels were sustained in the surgery group over time, whereas HbA1c levels increased in the non-surgery group.

In terms of overall trend, the surgery group experienced a sharp decline in HbA1c from baseline to Year 1, followed by a gradual upward trend from Years 2 to 5. In contrast, the non-surgery group showed only a slight decrease from baseline to Year 1, followed by a consistent rise in HbA1c thereafter. Across all time points the surgery group consistently maintained lower mean HbA1c values, and the differences between groups were statistically significant (Fig. 3).

## Discussion

This comparative cohort study highlights the real-world evidence supporting the substantial clinical benefits associated with metabolic and bariatric surgery for patients with obesity and type 2 diabetes mellitus. At five years follow-up the surgery group maintained 22.0% mean %TWL compared to 8.6% in the non-surgery group. In addition, 58.1% of patients in the surgery group achieved ≥20% TWL, compared to only 11.0% in the non-surgery group, and there was a fivefold higher likelihood of achieving optimal clinical response weight loss. The study finding that 11.0% of non-surgery patients achieved ≥20% TWL at 5 year follow-up is consistent with the study of Xie et al. who, using data from the US National Health and Nutrition Examination Survey (NHANES), reported that 15% of surgery-eligible individuals who did not undergo surgery still achieved ≥20% TWL [24]. A sub-analysis in the current study showed a trend of accelerated weight loss between years 2 and 3, suggesting possible delayed intervention (Supplementary Fig. S2), with the average %TWL in the non-surgery group increasing from 4.2% at one year to 8.6% at five years follow-up. This is in contrast to the typical pattern of weight loss commonly observed in non-surgical interventions where studies have shown that non-surgical strategies typically exhibit an initial weight reduction which is subsequently followed by a gradual weight regain over time [35, 36]. So while non-surgical strategies are generally associated with modest and less durable weight loss given the generally limited long-term effectiveness of lifestyle and medical weight loss interventions [15, 37], this was not the case for this cohort. Given that this sustained increase is unexpected, possible explanations here are that some patients may have received surgery elsewhere, there may be a highly motivated subset of patients who achieved and sustained a behaviour change or engaged with structured weight management programs and/or pharmacotherapy, or that only the most engaged patients remained in follow-up. Given the lack of intervention information, these findings should be interpreted with caution.

The multivariable mixed model findings indicate that in the surgery group %TWL decreases by an estimated 1.31% for each one-year increase in time, whereas in the non-surgery group it increases by an estimated 1.11%. While at first glance the estimated decrease in %TWL for the surgery group may be unexpected, it is consistent with established trajectories of postoperative weight regain after an initial 12–24 months [14, 38, 39], and a systematic review by Laut et al. also reported that between 5.7% and 75.6% of patients may experience recurrent weight gain within six years following bariatric surgery [38]. This is often attributed to behavioural factors (such as dietary habits, physical activity), anatomical changes (e.g. gastric pouch dilation), or metabolic adaptation [40]. The modest annual increase in %TWL (1.11%) estimated for the non-surgical group, consistent with the 11.0% in the non-surgery group who achieved ≥20% TWL at year 5, may again indicate that some patients may have received surgery elsewhere, or that there may be a highly motivated subset of patients, or that only the most engaged patients remained in follow-up.

Bariatric surgery also demonstrated significantly greater efficacy in glycemic control. At five years, 42.5% of surgery patients achieved HbA1c <6.0%, and 55.2% achieved ≤6.5% compared to only 7.8% and 13.9% respectively in the non-surgery group. Mean HbA1c reduction was 1.0% in the surgery group, while the non-surgery group showed a 0.4% increase. These results align with previous studies including the SurgiCal Obesity Treatment Study (SCOTS) and STAMPEDE, which demonstrated marked improvements in glycemic control post-surgery [41–44]. However, the magnitude of HbA1c reduction in our study was smaller than in SCOTS (2.7%) and STAMPEDE (2.1%) [41, 44]. These differences may be explained by differences in baseline characteristics, study design, and treatment intensity between the studies. For instance, STAMPEDE included patients with lower BMI (27–34 kg/m²) and provided intensive medical therapy to the control group unlike the non-surgery cohort in this study for whom post-referral care remains unknown.

The longitudinal analysis further confirmed a decline in HbA1c by an estimated 0.13% annually in the surgical group, contrasting with an estimated 0.11% annual increase in the non-surgical group. This may reflect the progressive nature of T2DM in the absence of intervention and highlights the unique metabolic effects of metabolic bariatric surgery, which includes improved insulin sensitivity, enhanced beta-cell function, and hormonal changes such as increased GLP-1 and PYY levels [9, 45, 46]. The findings of this study support the importance of surgical intervention for maximal glycemic benefit.

The study also revealed that 54.7% of eligible referred patients did not undergo metabolic and bariatric surgery, highlighting an important gap between eligibility and treatment uptake. This aligns with national trends in England, where ~7.3% of the adults meet the eligibility criteria for bariatric surgery, yet only about 0.2% receive the procedure annually [20]. Similarly, Alvarez et al. reported a 53.5% dropout rate in their study of 484 patients [21]. Several factors may explain this gap including challenges in meeting pre-operative requirements such as completing a formal weight management programme, achieving weight loss targets, or adhering to pre-surgical recommendations among others [21, 30, 47]. However, the specific reasons for non-uptake could not be determined in this study as this was beyond the scope of the data collected. A plausible contributing factor is the variation in the implementation of the National Institute for Health and Care Excellence (NICE) guidelines (NG246) across Integrated Care Systems (ICSs) and NHS health boards [48–50]. Some ICSs have introduced stricter criteria than those recommended by NICE, such as mandatory pre-surgery weight loss, specific comorbidities (Type 2 diabetes), higher BMI thresholds, or minimum durations of obesity, creating regional inconsistencies in access [48]. While there is no direct evidence, it is plausible that the eligibility criteria set by University Hospital Ayr may have influenced the results observed in this study.

Although bariatric surgery is considered a cost-effective treatment for severe obesity and T2DM [51, 52], its benefits may not be fully realised in this population due to limited or delayed uptake [53]. This has both clinical and economic implications. Delays in bariatric surgical intervention may worsen obesity-related comorbidities, increase healthcare utilisation, and lead to indirect societal costs associated with reduced productivity and long-term care requirements [26, 53].

The disparity in the implementation of NICE guideline (NG246) highlights the need for a standardised national framework to ensure consistent application and equitable access to bariatric surgery across all regions [50].

### Strength and limitations

A major strength of this study lies in its use of real-world clinical data with a five-year follow-up. The comparison between surgical and non-surgical patients within the same referral cohort provides important insights into routine patients care and complements the findings of randomized controlled trials. However, the study also has limitations. First, the presence of missing data for key outcomes such as weight and HbA1c. To mitigate this the study employed repeated measures analysis, a method that is flexible in handling missing values by using all available data from the observations. Secondly, there was a lack of detailed data regarding post-referral care particularly among individuals in the non-surgery group. This limitation may have influenced the study findings as it remains unclear whether these individuals participated in structured medical weight management programs, received intensive lifestyle interventions, or were managed through standard care. Therefore, the interpretation of the results should be undertaken with caution, considering this uncertainty.

Additionally, important factors known to influence bariatric surgery outcomes, such as dietary habits, physical activity, and adherence, were not available from the electronic databases. The absence of these variables limited the ability to comprehensively assess their influence on outcomes.

## Conclusion

The current study finds that patients in the surgery group achieved significantly higher %TWL and HbA1c reduction compared to non-surgery group patients, which aligns with a wide body of research and highlights the role of bariatric surgery as the most effective intervention for sustained weight loss and glycemic control. Importantly, the findings highlight the need for structured, long-term follow-up to support sustained outcomes, particularly in mitigating gradual recurrent weight gain and ensuring continued glycemic control benefit. Also, given the demonstrated advantages of bariatric surgery and the progressive nature of type 2 diabetes, timely surgical intervention should be prioritised for eligible patients. These results support current clinical guidelines that recommend metabolic bariatric surgery for individuals with severe obesity or obesity-related complications who do not achieve adequate results through more conservative treatments. Future studies should investigate the reasons why eligible patients choose not to undergo bariatric surgery, despite its potential benefits. Research should also examine the broader burden of disease, including the incidence and progression of obesity-related comorbidities, and the associated healthcare costs among both surgical and non-surgical patients. Such studies would provide valuable insight into the economic and clinical benefits of bariatric surgery, thereby informing cost-effectiveness evaluations and healthcare policy decisions.

## Supplementary information

Sup_Tables_Figures.

## Data Availability

The datasets generated and/or analysed during the current study are not publicly available because of data sharing agreements and restrictions associated with the use of electronic health record data.
