# Supplementary material for: Metabolic bariatric surgery pays off: a longitudinal analysis of weight loss and HbA1c changes in real-world patients data in the West of Scotland
Source: Int J Obes (Lond). 2025 Nov 19;50(3):527–35. doi: 10.1038/s41366-025-01956-6 (PMC12965875; doi:10.1038/s41366-025-01956-6)
Supplement: Supplementary file 1 — Sup_Tables_Figures. [file 41366_2025_1956_MOESM1_ESM.docx]

**Supplementary Tables and Figures**

Table S1 Adjusted Estimates of achieving optimal clinical response weight Loss at Five Years.

| **Outcomes** | **Non-surgery group** | **Surgery group** |
| --- | --- | --- |
| **Achieved ≥20 %TWL** |  |  |
| ^a^Proportion achieved ≥20 %TWL | 10.7% (4.78 – 16.83) | 59.4% (50.97 – 67.84) |
| ^a^Multivariable adjusted prevalence ratio | Ref | 5.49 (3.08 – 9.89) |
| ^a^Adjusted for baseline age, sex, Scottish Index of Multiple Deprivation quintiles, pre-surgery BMI  %TWL=Percentage Total weight loss | | |

Table S2 Estimated %TWL with 95% CI, comparison between surgery and non-surgery group from linear mixed model.

| **Follow-up time** | **Year 1** | **Year 2** | **Year 3** | **Year 4** | **Year 5** |
| --- | --- | --- | --- | --- | --- |
| **N** | 358 | 328 | 273 | 233 | 229 |
| Surgery  Mean (95% CI) | 26.5 (25.3 - 27.8) | 25.8 (24.6- 27.0) | 23.2(21.8 - 24.5) | 22.2(20.5- 23.8) | 22.0(20.1- 23.9) |
| Non-surgery  Mean (95% CI) | 4.2 (3.0 – 5.4) | 4.1 (2.8 - 5.3) | 6.1(4.6- 7.4) | 7.2(5.6- 8.8) | 8.6 (6.6- 10.5) |
| n = surgery | n=178 | n=178 | n=150 | n=113 | n=129 |
| n = non-surgery | n=180 | n=149 | n=123 | n=120 | n=100 |


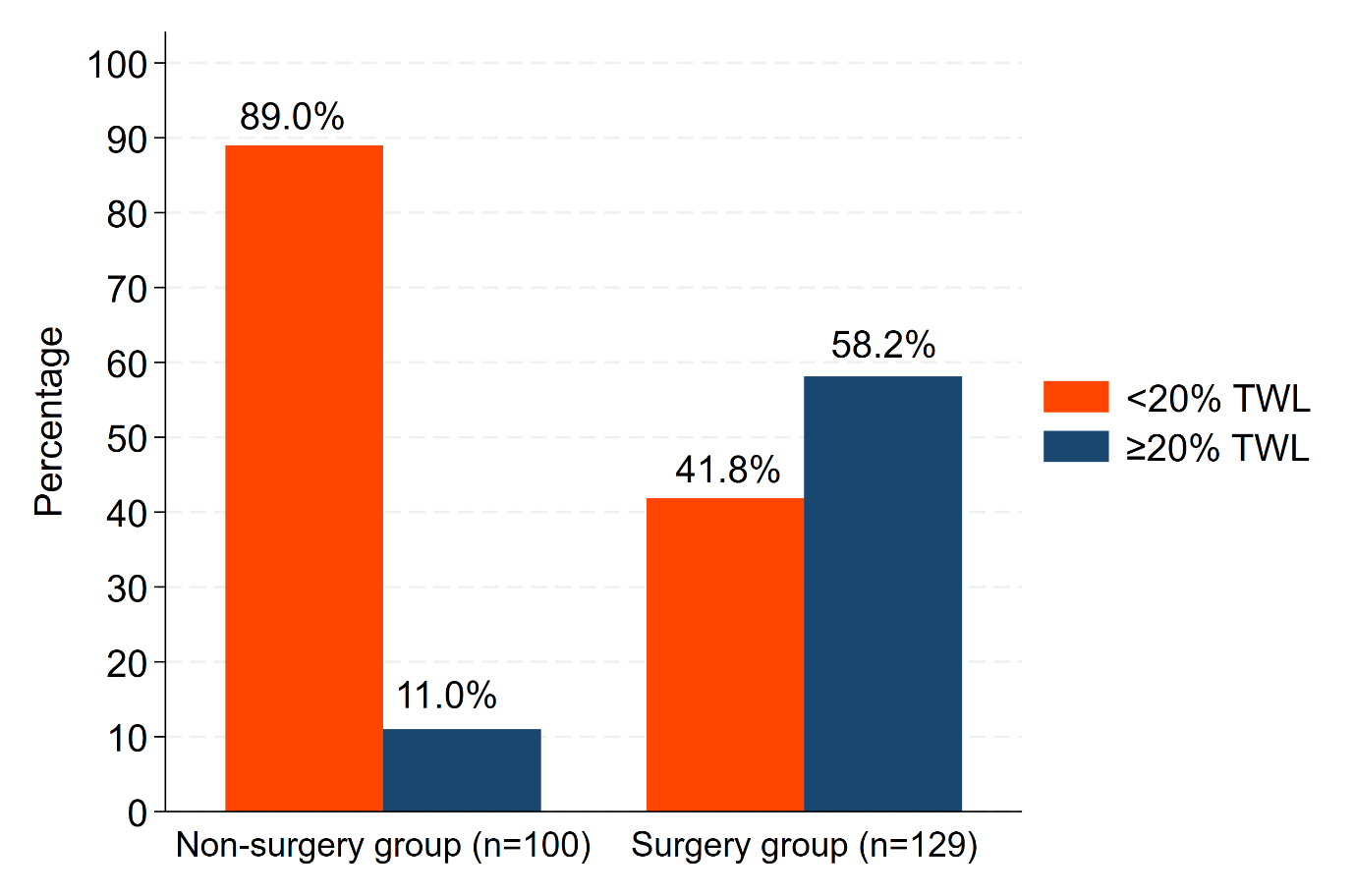


Figure S1 Proportion of patients who achieved ≥20% total weight loss at five years.


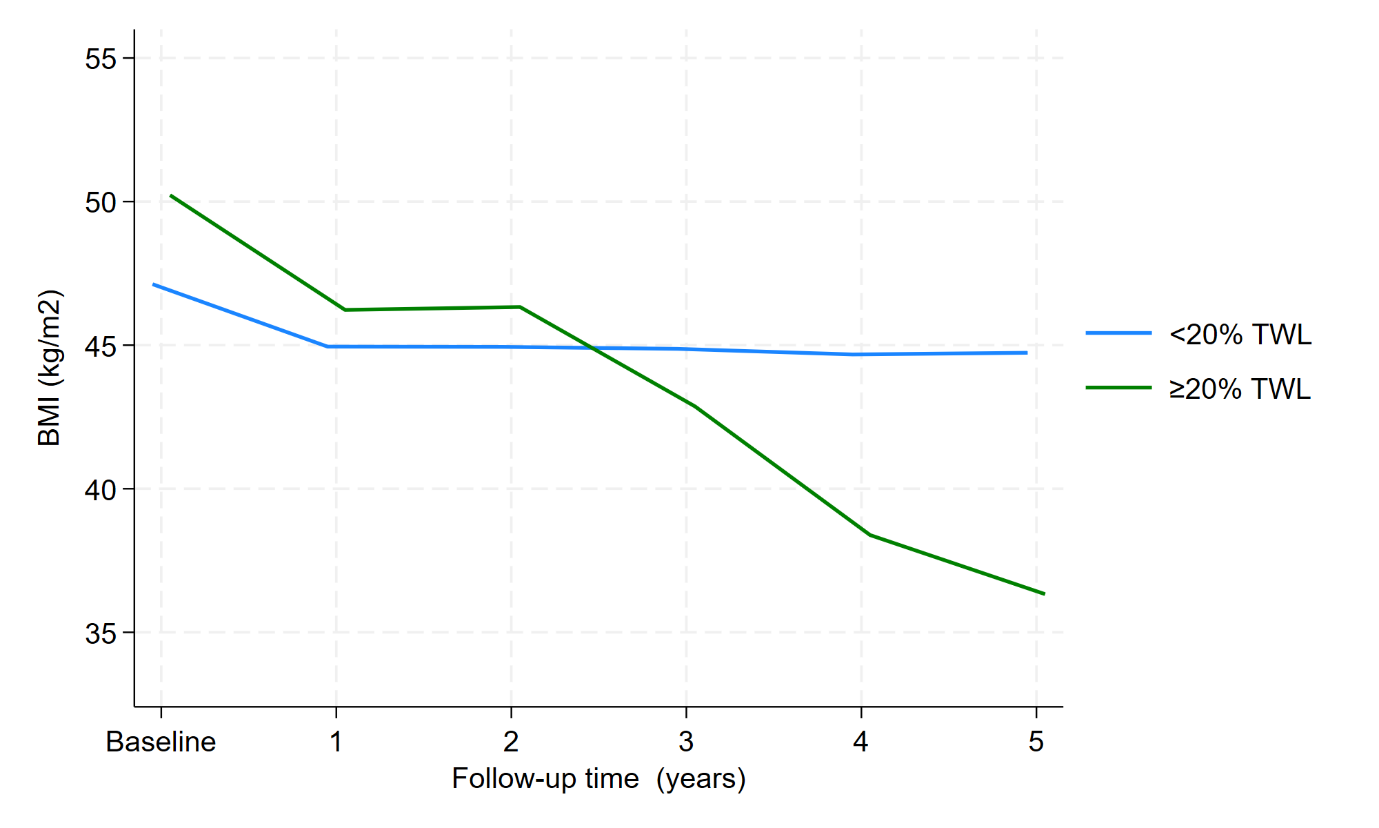


Figure S2 Line graph of Mean BMI across different time points according to patients’ optimal clinical response weight loss status in non-surgery group.
